# A Role for BK Channels in Heart Rate Regulation in Rodents

**DOI:** 10.1371/journal.pone.0008698

**Published:** 2010-01-14

**Authors:** Wendy L. Imlach, Sarah C. Finch, John H. Miller, Andrea L. Meredith, Julie E. Dalziel

**Affiliations:** 1 AgResearch, Grasslands Research Centre, Palmerston North, New Zealand; 2 AgResearch, Ruakura Research Centre, Hamilton, New Zealand; 3 School of Biological Sciences, Victoria University of Wellington, Wellington, New Zealand; 4 Department of Physiology, University of Maryland School of Medicine, Baltimore, Maryland, United States of America; 5 Department of Pharmacology and Toxicology, University of Otago, Dunedin, New Zealand; University of Cincinnati, United States of America

## Abstract

The heart generates and propagates action potentials through synchronized activation of ion channels allowing inward Na^+^ and Ca^2+^ and outward K^+^ currents. There are a number of K^+^ channel types expressed in the heart that play key roles in regulating the cardiac cycle. Large conductance calcium-activated potassium (BK) ion channels are not thought to be directly involved in heart function. Here we present evidence that heart rate can be significantly reduced by inhibiting the activity of BK channels. Agents that specifically inhibit BK channel activity, including paxilline and lolitrem B, slowed heart rate in conscious wild-type mice by 30% and 42%, respectively. Heart rate of BK channel knock-out mice (*Kcnma1^−/−^*) was not affected by these BK channel inhibitors, suggesting that the changes to heart rate were specifically mediated through BK channels. The possibility that these effects were mediated through BK channels peripheral to the heart was ruled out with experiments using isolated, perfused rat hearts, which showed a significant reduction in heart rate when treated with the BK channel inhibitors paxilline (1 µM), lolitrem B (1 µM), and iberiotoxin (0.23 µM), of 34%, 60%, and 42%, respectively. Furthermore, paxilline was shown to decrease heart rate in a dose-dependent manner. These results implicate BK channels located in the heart to be directly involved in the regulation of heart rate.

## Introduction

Large conductance calcium-activated potassium (BK) ion channels are expressed in many tissues that exhibit diverse physiological characteristics. BK channels are activated by intracellular calcium and depolarizing membrane voltages. BK channels are highly expressed in smooth muscle where they have been shown to affect myogenic tone [1] and therefore regulate blood pressure [2], cerebrovascular circulation [3], erectile function [4], and urinary bladder function [5], [6]. They are also prevalent in the brain where they have important roles in the regulation of neuronal circuits in the hippocampus [7], in motor function and cerebellum [5], [8], [9], and in circadian rhythm and the hypothalamus [10]. BK channels have also been found to have roles in hearing [11], [12], kidney filtration [13], colonic K^+^ secretion [14], [15], and immune function [16]. BK-type channels are thought to be present in the inner mitochondrial membrane of cardiac myocytes and protect against cardiac ischemia [17].

The BK channel α-subunit assembles as a homotetramer to form the channel pore [18], [19]. It may associate with an accessory β subunit(s) of which four have been identified, β1, β2, β3 and β4, that have restricted expression patterns and modify channel activity by altering sensitivity to voltage and calcium, or affect inactivation (for review see [20]). The β1 subunit is most highly expressed in smooth muscle, β2 in the ovary, β3 in the testis, and β4 subunit in neural tissue [21], [22], although lower levels exist in other tissues, and some β subunits appear to coexist [22], [23].

The molecular function of BK channels has been well studied and has been greatly aided by the use of BK channel inhibitors, for example, tetraethylammonium (TEA), the peptide inhibitors charybdotoxin and iberiotoxin, and the fungal alkaloids paxilline and lolitrem B. Iberiotoxin is the most specific BK channel inhibitor yet characterized [24], [25], [26], but it is of low activity against channels containing the β4 subunit [27], [28], and is membrane-impermeable, limiting its use in whole animal experiments. The membrane-permeable fungal alkaloid, paxilline has become widely used as a BK channel inhibitor in molecular physiology due to its ability to block BK channels complexed with β4 subunits [29]. More recently, however, another fungal alkaloid, lolitrem B has been shown to be five-times more potent at inhibiting BK channels in comparison to paxilline [30], [9]. A further seven lolitrem compounds have also been shown to be BK channel inhibitors [31]. Lolitrem B is the causative agent of ryegrass staggers, a nervous disorder of animals that graze perennial ryegrass infected with the endophytic fungi *Neotyphodium lolii*. Using a mouse model of ryegrass staggers, we have shown that lolitrem B produces ataxia and tremors by inhibiting BK channels [9]. In addition to lolitrem B, this endophyte-grass symbiosis also produces other structurally related lolitrem analogues in which only minor structural changes have a dramatic effect on tremorgenicity [32], [33], [34].

As part of a study on the physiological effects of lolitrem B and paxilline in mice we monitored cardiovascular function and observed unexpected responses to these compounds. Since BK channels expressed in smooth muscle regulate vascular tone [1], [2], it may be anticipated that BK channel inhibitors might affect blood pressure when administered to mice. Contrary to this, however, we found no change in blood pressure but observed a dramatic effect on heart rate. This effect was surprising since there was no published evidence of a direct role for BK channels in the regulation of heart rate. However, we note that there are only a few reports in the literature on the effects of BK channel inhibitors in whole animals [35], [36], and none designed to study their effects on heart rate.

Although heart beat initiation and propagation involves the interplay among many ionic currents from a range of ion channels, electrophysiological studies of BK channels in the cardiac muscle plasma membrane show no evidence of BK channel involvement, and given the low level of BK channels expressed in the whole heart, a role of these channels in heart function has previously been overlooked [37], [38], [39], [40], [41]. Heart rate is initiated in the sinoatrial (SA) node pacemaker and is modulated by autonomic inputs: parasympathetic cholinergic neurons decrease heart rate via the vagus nerve, and sympathetic adrenergic neurons increase heart rate. Functional BK channels are present in these autonomic neurons and contribute to action potential repolarisation and after-hyperpolarisation currents [42], [43]. Although BK channels are present in Purkinje fibres of the cardiac ventricle conduction system [44], any effects from this location would be downstream of that from heart beat initiation and would therefore not be expected to alter rate. Although BK channels have also been identified in the inner mitochondrial membrane of myocytes [17], it is unlikely that their action at this site would influence pacemaker function.

The aim of this study was to investigate the role of BK channels in cardiovascular function. The BK channel inhibitors lolitrem B, paxilline, and iberiotoxin were used as pharmacological tools to investigate effects of loss of BK channel function on blood pressure and heart rate in wild-type mice and results compared with that in BK channel knock-out mice. To determine whether the BK channel antagonist effects were cardioselective or worked at peripheral sites, we also investigated BK channel function in an isolated, perfused rat heart preparation.

## Materials and Methods

### Animals

#### Mice

BK channel–null (*Kcnma1^−/−^*) mice had an FVB background, and their wild-type littermates were used as controls [5]. Heterozygous mice were used as breeding pairs, and all mice were genotyped by PCR. The β1- and β4-subunit-null (*Kcnmb1^−/−^* and *Kcnmb1/b4^−/−^*) mice [2], [7] had a mixed C57BL/6J background, and C57BL/6J wild-type mice were used as strain controls. Male and female mice aged between 2 and 24 months were used, and treatment groups were matched for age and sex.

#### Rats

Male and female Sprague Dawley rats between the ages of 3 and 4 months were used for Langendorff isolated heart experiments. Animals were asphyxiated by carbon dioxide inhalation and decapitated prior to removal of the heart for perfusion.

### Compounds

Lolitrem B and lolitrem E were extracted and purified from perennial ryegrass infected with *N. lolii* as previously described [32]. Lolitrem E acetate was synthesized from lolitrem E [45] and 31-*epi*lolitrem B from lolitrem B [34]. Paxilline was produced by *Penicillium paxilli* cultures as described previously [46]. rIberiotoxin was purchased from Alomone Labs, Product # RTI-400 (Jerusalem, Israel).

### Ethics Approval

Animal manipulations were approved by the AgResearch Ruakura Animal Ethics Committee (NZ), Victoria University Animal Ethics Committee (NZ) (permission given to euthanize animals for tissue harvest) and the Stanford University Animal Care and Use Committees (USA).

### Blood Pressure Analysis in Mice

Mean blood pressure and heart rate were measured in conscious animals with a blood pressure analysis system utilizing a tail-cuff method (BP-2000, Visitech Systems). Mice were trained for 3 consecutive days in the pre-warmed (30°C) device to avoid a stress-induced increase in blood pressure. For each blood pressure determination, 10 measurements were obtained and averaged per mouse.

### Langendorff Preparation – Isolated, Perfused Rat Heart

The use of the Langendorff rat heart preparation has recently been reviewed [47]. To set up a standard, non-working heart preparation, a rat was partially asphyxiated with CO_2_ then decapitated and the heart removed following injection of 30 µl heparin (16 U/ml saline) into the inferior vena cava. The aorta was cannulated, and the coronary circulation perfused retrogradely by gravity feed (78 cm height) with 37°C oxygenated (95% O_2_, 5% CO_2_ – Carbogen, BOC gases, Lower Hutt, NZ) Krebs-Henseleit solution (118.5 mM NaCl, 25.0 mM NaHCO_3_, 4.7 mM KCl, 1.2 mM MgSO_4_, 1.2 mM KH_2_PO_4_, 11.0 mM glucose, 1.8 mM CaCl_2_, pH 7.4). A PowerLab system (ADInstruments, Model 8SP, Dunedin, NZ) was used to monitor cardiac function with inputs supplied from a pressure transducer connected in-line with the aortic cannula (Medstad, model no. 60–800) and three electrocardiographic leads attached to the apex of the heart (two leads) and lower left ventricle (one lead) to measure the ECG. Immediately after perfusion commenced, the coronary vessels cleared of blood, and the heart began to beat strongly within a few seconds. Heart rate and aortic pressures were continuously recorded, beginning with an equilibration period of at least 40 min, and analyzed using Chart5 for Windows (v5.2.2, ADInstruments). Heart temperature was continuously monitored with a scanning tele-thermometer fitted with a needle thermistor (Yellow Springs Instrument Co., Model 47, Yellow Springs, OH) and temperature was maintained at 37±0.5°C throughout the experiment. Coronary flow was measured by collecting the perfusate outflow over time.

### Drug Delivery

Whole mouse experiments: Toxins were administered to mice by intraperitoneal injection as a solution in 9∶1 (v/v) DMSO-water (50 µl).

Isolated heart: Drugs were administered to the isolated heart by intracoronary infusion through the aortic cannula using a syringe pump (KD Scientific, model KDS120). The drug infusion speed was set at 1 ml/min, about 10% of normal coronary flow rate for an isolated rat heart. To control for the diluent needed to solubilize lolitrem B and paxilline, 0.1% DMSO in Krebs-Henseleit buffer was infused into the aortic cannula for 12 min, followed by 30 min monitoring of cardiac activity. Isolated hearts were treated with 0.23 µM rIberiotoxin, or 1–10 µM paxilline, as described above. Dose-response experiments were not performed for rIberiotoxin or lolitrem B due to the high cost of these compounds.

### Statistical Analyses

Results where *n*≥3 are expressed as means. Error bars in each figure show ± S.E.M. Treatments were compared using a least significant post hoc test after analysis of variance except when there were four measurements made within each mouse in which case a repeated measures one-way analysis of variance followed by Tukey-Kramer multiple comparisons post hoc test was used. Between animal differences were accounted for in all tests. A paired Student's t-test using was also used. InStat version 3.0b (GraphPad Software, Inc) and Genstat version 11.1 (VSN International Limited, Hemel Hempstead, UK) software was used.

## Results

### Inhibition of BK Channels *In Vivo* Has No Effect on Blood Pressure but Decreases Heart Rate

In order to investigate the contribution of BK channels to cardiovascular function, effects of two indole diterpene BK channel inhibitors, lolitrem B and paxilline, were examined. Both compounds were used because of differences in their potencies and duration of effect *in vivo* [48], [9]. Dose rates of 4 mg/kg lolitrem B and 8 mg/kg paxilline were chosen since these levels produce significant motor function deficits in wild-type mice [9]. The compounds were administered to mice and their effects on blood pressure and heart rate measured.

**Figure 1:**
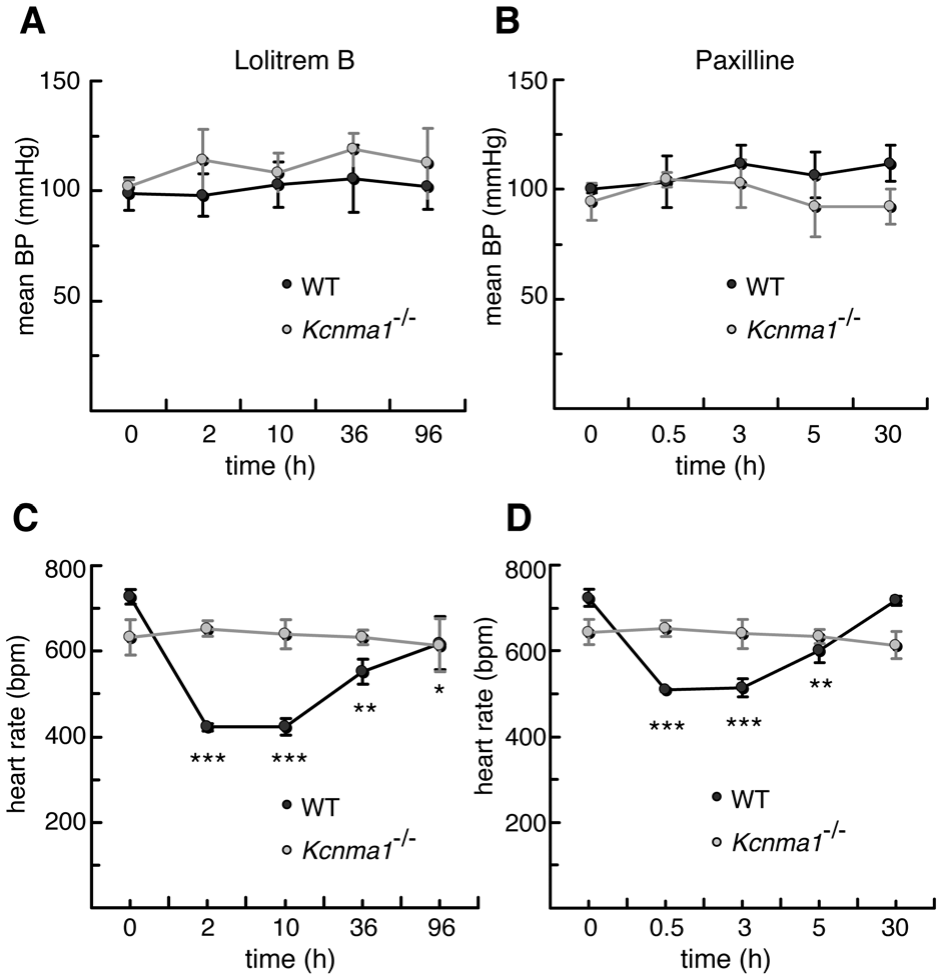
The effects of lolitrem B and paxilline on blood pressure and heart rate in *Kcnma1* ^−/−^ mice and wild-type littermates. Inhibitors were added immediately after the control reading at time zero as indicated by the arrow. Mean blood pressure following treatment with (A) 4 mg/kg lolitrem B, or (B) 8 mg/kg paxilline. Heart rate following treatment with (C) lolitrem B, or (D) paxilline. Data are mean ± S.E.M. for *n* = 4 in each treatment group. Significance was tested using Tukey-Kramer post hoc test after a repeated measures analysis of variance. Asterisks indicate the significance of each treatment in wild-type mice compared with the pre-drug control at time = 0. *, P<0.05; **, P<0.01; ***, P<0.001.

Both lolitrem B and paxilline were found to have no effect on mean blood pressure in wild-type mice (Fig. 1A & B), but both inhibitors induced a significant decrease in heart rate (Fig. 1C & D). In wild-type mice treated with paxilline, heart rate was decreased by 31% 30 min after toxin administration, an effect that lasted for 3 h, followed by a gradual recovery to baseline levels by 30 h (Fig 1D). In mice dosed with lolitrem B, heart rate was decreased by 42% 2 h after treatment which persisted for a further 8 h, followed by a slow recovery over 4 days (Fig 1C). The difference in time-course observed for paxilline and lolitrem B is consistent with that observed for the motor function effects [9]. These results show that BK channel inhibitors significantly decrease heart rate under conditions of normal autonomic tone.

To determine whether the observed heart rate effects also occurred with structurally related compounds of varying potency, we extended the study to include a further two lolitrem compounds. 31-*epi*lolitrem B is non-tremorgenic in mice [34] and is less potent as a BK channel inhibitor (IC_50_ = 50 nM) in comparison to paxilline (IC_50_ = 22 nM) and lolitrem B (IC_50_ = 4 nM) [31]. Lolitrem E acetate is the most potent BK channel inhibitor of this structural class (IC_50_ = 2.6 nM) [31], but induces only low intensity tremors at high concentrations in mice [32]. The effects of these non-tremorgenic BK channel inhibitors are shown in Figure S1. Lolitrem E acetate (4 mg/kg) decreased heart rate by 30% but had no effect on blood pressure. 31-*epi*lolitrem B (20 mg/kg) had no effect on heart rate or blood pressure. Since lolitrem B was the most potent lolitrem to affect heart rate, it was used in subsequent experiments. Paxilline was also used because of its relatively short onset and rapid recovery.

### *Kcnma1* ^−/−^ Mice Are Insensitive to Cardiovascular Effects of BK Channel Inhibitors

To determine whether the heart rate decrease induced by lolitrem B and paxilline is specifically due to BK channel inhibition, we examined the effects of both compounds on mice with a targeted deletion in the gene encoding the pore-forming unit of the BK channel (*Kcnma1* ^−/−^). Lolitrem B had no effect on blood pressure or heart rate in *Kcnma1* ^−/−^ mice (Fig 1A & C), and paxilline was similarly without effect (Fig 1B &1D). A drug vehicle control of DMSO∶water (9∶1) was administered which showed no change in blood pressure or heart rate in either wild-type or *Kcnma1* ^−/−^ mice. These results indicate that the BK channel α subunit is required for lolitrem B and paxilline to reduce heart rate in mice and provide further support for a specific role of BK channels in heart rate control.

### Paxilline-Mediated Effects on Heart Rate Do Not Involve the β1 or β4 Subunits

Given the functional heterogeneity that is conferred on BK channels by the accessory beta subunits, we investigated whether β1 and β4 subunits are required for the cardiovascular effects of the BK channel inhibitors. The β1 subunit is highly expressed in blood vessels, including the coronary arterioles, and therefore it was considered that its role in blood pressure regulation might indirectly affect heart rate [49]. A possible role for the β4 subunit in regulation of heart rate was investigated because it is expressed in neural tissue and is likely to be present in the neurons that innervate the heart [22]. An effect that requires the β4 subunit would indicate that the channels containing this subunit are involved in regulation of heart rate, and that the effect is likely of neural origin.

**Figure 2:**
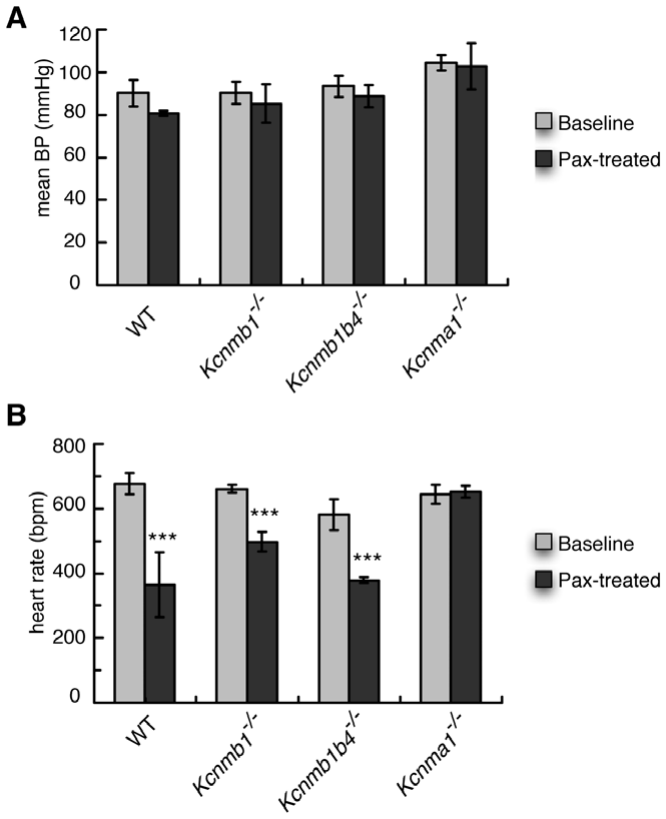
Cardiovascular function in wild-type (C57 black), *Kcnmb1^−/^* ^−^, *Kcnmb1/b4^−/−^*, and *Kcnma1^−/−^* mice treated with 8 mg/kg paxilline (Pax). (A) Blood pressure and (B) Heart rate. ***, P<0.001 for significant differences between pre-treated and paxilline-treated heart rates. Significance was tested using least significant difference post hoc test after analysis of variance. All data are mean ± S.E.M. for *n* = 4 in each treatment group.

To determine whether the β1 or β4 subunits are involved in the BK channel-mediated chronotropic effects, β1 and β1/β4 knock-out mice (*Kcnmb1^−/−^* and *Kcnmb1b4^−/−^*, respectively) were treated with 8 mg/kg paxilline and heart rate and blood pressure monitored. Paxilline had no effect on the blood pressure of *Kcnmb1^−/−^* and *Kcnmb1b4^−/−^* knockout mice, as seen in wild-type controls (Fig. 2B). However, a substantial decrease in heart rate was detected in response to paxilline in *Kcnmb1^−/−^* and *Kcnmb1b4^−/−^* knockout mice, similar to that seen in wild-type mice. This suggests that the BK channel β1 and β4 subunits are not required for paxilline to decrease heart rate.

### Inhibition of BK Channels in the Isolated, Perfused Rat Heart Decreases Heart Rate

**Figure 3:**
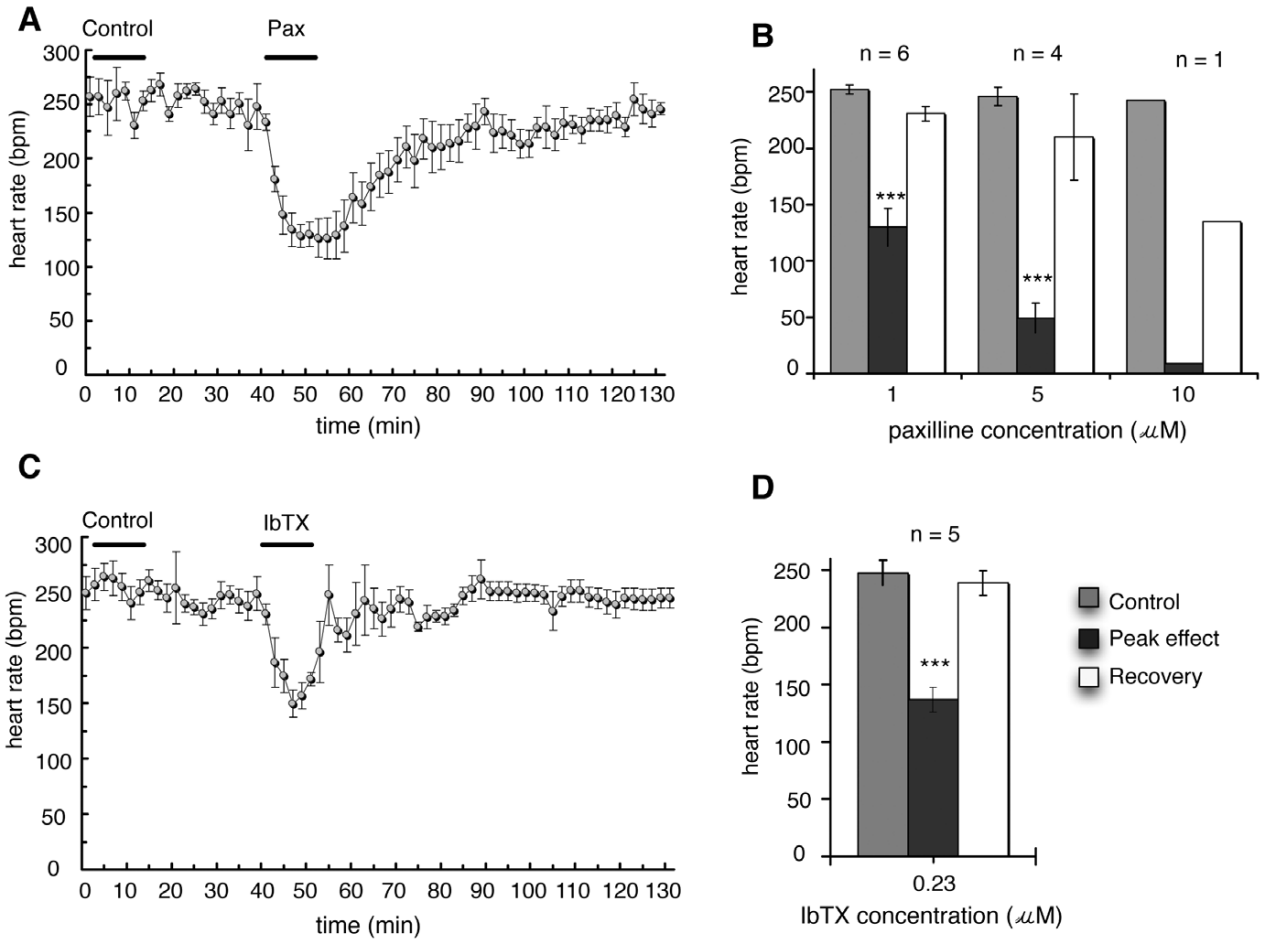
The effect of paxilline and iberiotoxin on heart rate in the isolated rat heart. (A) 1 µM paxilline (Pax) was added at time = 40 min and infused for 10 min, preceded by a control infusion of Krebs-Henseleit perfusion fluid with 0.1% DMSO (*n* = 5). (B) Heart rate before, during and after infusion at time zero of different concentrations of paxilline (1, 5 or 10 µM). The heart rate in response to paxilline is the average of the peak responses following treatment. Toxins dissolved in Krebs-Henseleit solution were perfused through the heart for 12 min. Significance was tested using least significant difference post hoc test after analysis of variance. (C) Heart rate of isolated, perfused rat hearts infused with 0.23 µM iberiotoxin (IbTX) following a control infusion of Krebs-Henseleit fluid (n = 5). (D) Heart rate before treatment (saline control), at peak effect following infusion of iberiotoxin (n = 5), and following recovewry. Significance was tested using a paired Student's t-test. All data are mean ± S.E.M. *** P<0.001.

To determine whether the effects on heart rate were mediated by a direct effect on the heart or through other secondary cardiovascular effects, experiments were performed using isolated, perfused rat hearts. Each heart was monitored for a minimum of 30 min, during which time a vehicle control was perfused for 10 min, prior to adding the BK channel inhibitor. The perfusion fluid contained ≤0.1% DMSO to help solubilize the indole diterpenoid compounds, as used in previous *in vitro* experiments [30], [9], [31]. Paxilline (1 µM) infusion over 10 min induced a decrease in heart rate of 34% at 5 min, an effect which lasted for a total of 20 min. Heart rate then returned to baseline levels over the next 30 min (Fig. 3A). Lolitrem B was also tested at 1 µM and was found to decrease heart rate by 60% (recording not shown). Lolitrem B had a long latency of 90–120 min before heart rate began to decrease, and recovery to baseline levels was not observed even after a further 60 min of drug-free perfusion. This extended the time beyond the viable life-span of the preparation, and, therefore, longer recovery experiments were not pursued.

To determine the maximum level of heart rate decrease that could be induced by inhibition of BK channels, increasing concentrations of paxilline were tested using a new heart for each trial at each concentration. Paxilline concentrations of 1, 5 and 10 µM were tested which resulted in heart rate decreases of 34%, 70% and 96%, respectively (Fig. 3B).

Since paxilline showed a strong bradycardic effect in the isolated heart, we wanted to compare this effect with another BK channel inhibitor. Iberiotoxin (IbTX) was used and tested in a similar manner since it is highly specific for BK channels [24], [25], [26]. In initial experiments, low concentrations of IbTX were applied to isolated hearts until a concentration was reached that gave a heart rate decrease. This concentration (0.23 µM) was used in all subsequent experiments. IbTX decreased heart rate by 42% over 5 min, and this recovered to the predrug level over 15 min (P<0.001) (Fig. 3C). Perfusion of a DMSO vehicle control performed in each experiment had no effect on baseline heart rate (Fig. 3).

## Discussion

The decrease in heart rate produced by BK channel inhibitors, paxilline and lolitrem B, in wild-type mice was unexpected since BK channels are not thought to be present in the heart. The observation that these compounds had no effect on heart rate in *Kcnma1* ^−/−^ mice further implicates BK channels in this effect. The lack of effect of the BK channel inhibitors on blood pressure, using a non-invasive blood pressure analysis system, was supported by preliminary results obtained using telemetry equipment in which paxilline given to telemetrized, freely moving, wild-type mice (n  =  4, data not shown) also resulted in a decrease in heart rate. These results indicate that the decrease in heart rate is unlikely to be due to a secondary reflex compensatory effect related to a change in blood pressure since no immediate change in blood pressure was detected. It is unclear why no change in blood pressure was detected, given the contractile responses reported for BK channel inhibitors in smooth muscle *in vitro* [50], [51]. It is possible that this is due to redundancy among K^+^ ion channels in control of blood pressure. The normal blood pressure in *Kcnma1^−/−^* mice is consistent with that previously reported for this phenotype at rest [52].

The finding that BK channel inhibitors also induced bradycardia in isolated hearts supports the hypothesis that this effect is mediated through a direct effect of BK channel inhibitors on cardiac channels rather than indirectly through non-cardiac pathways. Of the inhibitors used, paxilline is the most commonly used in functional studies due to its membrane permeability and selectivity for BK channels, as non-specific effects have only been reported at high micromolar concentrations [53], [54]. The use of the highly specific BK channel inhibitor iberiotoxin [24], [25], [26] in addition to lolitrem B and paxilline strengthens the hypothesis that a BK channel is involved in the bradycardic response.

The slow onset of the bradycardic effect for lolitrem B compared with paxilline is also seen for their motor function effects [48], [9]. This delay, however, does not occur in macropatch recordings [30], [9], [31], suggesting that it relates to the ability of these compounds to access their binding sites *in vivo*.

Experiments using beta subunit knock-out mice strongly suggests that neither β1 nor β4 accessory subunits are required for paxilline to affect heart rate. This suggests that the bradycardia produced by these compounds is unlikely to be due to secondary vascular effects or neural regulation, since β1 or β4 subunits are required for the calcium-mediated vascular and neural effects, respectively. Sensitivity to iberiotoxin is greatly reduced in neural BK channels co-assembled with β4 subunits [27], [28]. The fact that iberiotoxin was able to decrease heart rate significantly in the isolated heart suggests that this effect at least partially involves BK channels that lack β4 subunits. Preliminary experiments conducted in the presence of the cholinergic antagonist atropine (3 µM) showed no change in the bradycardic effect of paxilline (1 µM) (data not shown). Atropine would limit any residual vagal cholinergic influence on the pacemaker that might decrease heart rate. While this preliminary observation suggests that autonomic involvement is unlikely, this possibility requires further investigation.

BK channels in coronary arterioles are a possible site of action for the inhibitors. However, coronary flow remained constant throughout the isolated rat heart experiments, indicating that coronary resistance was constant, and therefore vasoconstriction did not occur. Furthermore, potent vasoconstrictors have little or no effect on heart rate in the isolated heart [55], [56], [57], suggesting that any inhibition of BK channels in coronary blood vessels would be unlikely to make a substantial contribution to the observed bradycardia.

A pacemaker location for BK channels is a possibility that would enable direct regulation of heart rate. Although ion channel expression in the mouse pacemaker has been thoroughly investigated, no studies have examined BK expression in sinoatrial node (SAN) cells [58], [41]. BK channel mRNA expression has been quantified in the whole heart, but only low levels have been detected [38], [21], [40]. If BK channels are expressed solely in pacemaker cells, this might account for the low level of expression when the whole heart is examined, as the signal would be greatly diluted. Expression of BK channels at this site could easily go undetected because of the small area involved and difficulty in identifying the exact region where the SAN cells are located. From a pacemaker location BK channels might influence heart rate by modulating action potential firing and repolarisation. Alternatively, if BK channels are present in SAN cell mitochondria, their inhibition might influence heart rate through metabolic mechanisms.

It may seem surprising that chronotropic effects of BK channel inhibitors have not been reported previously. Several studies have examined paxilline effects in an isolated heart in the context of ischemia/reperfusion [17], [59], [60], but none examined the effect of paxilline on heart rate. Our study is the first to have examined the effect of BK channel inhibitors on heart rate by monitoring drug effects within animals, and in the isolated heart, using a range of paxilline concentrations. This may explain why we were able to observe a bradycardic effect that has not been previously reported.

The results presented in this study support the hypothesis that BK channels are expressed and functional in the heart. Their tissue location and role in cellular excitability remain to be determined. The bradycardic effects that BK channel inhibitors have on heart rate suggests that these channels could be potential targets for pharmacological modulation of heart rate for medicinal purposes, such as in hypertension, or during surgery.

## Supporting Information

Figure S1 Comparison of peak effect of indole diterpene compounds on heart rate and blood pressure in wild-type mice. The effect of 8 mg/kg paxilline (pax), 4 mg/kg lolitrem B (lol B), 8 mg/kg lolitrem E acetate (lol E ac), 20 mg/kg 31-epilolitrem B (31-epilol B), and DMSO controls, in wild-type mice on: (A) heart rate, and (B) blood pressure. Significance was tested using least significant difference post hoc test after analysis of variance. All data are mean ± S.E.M. *** P<0.001.
(4.85 MB TIF)
