# Supplementary figures and images for: A Role for BK Channels in Heart Rate Regulation in Rodents
Source: PLoS One. 2010 Jan 14;5(1):e8698. doi: 10.1371/journal.pone.0008698 (PMC2806827; doi:10.1371/journal.pone.0008698)

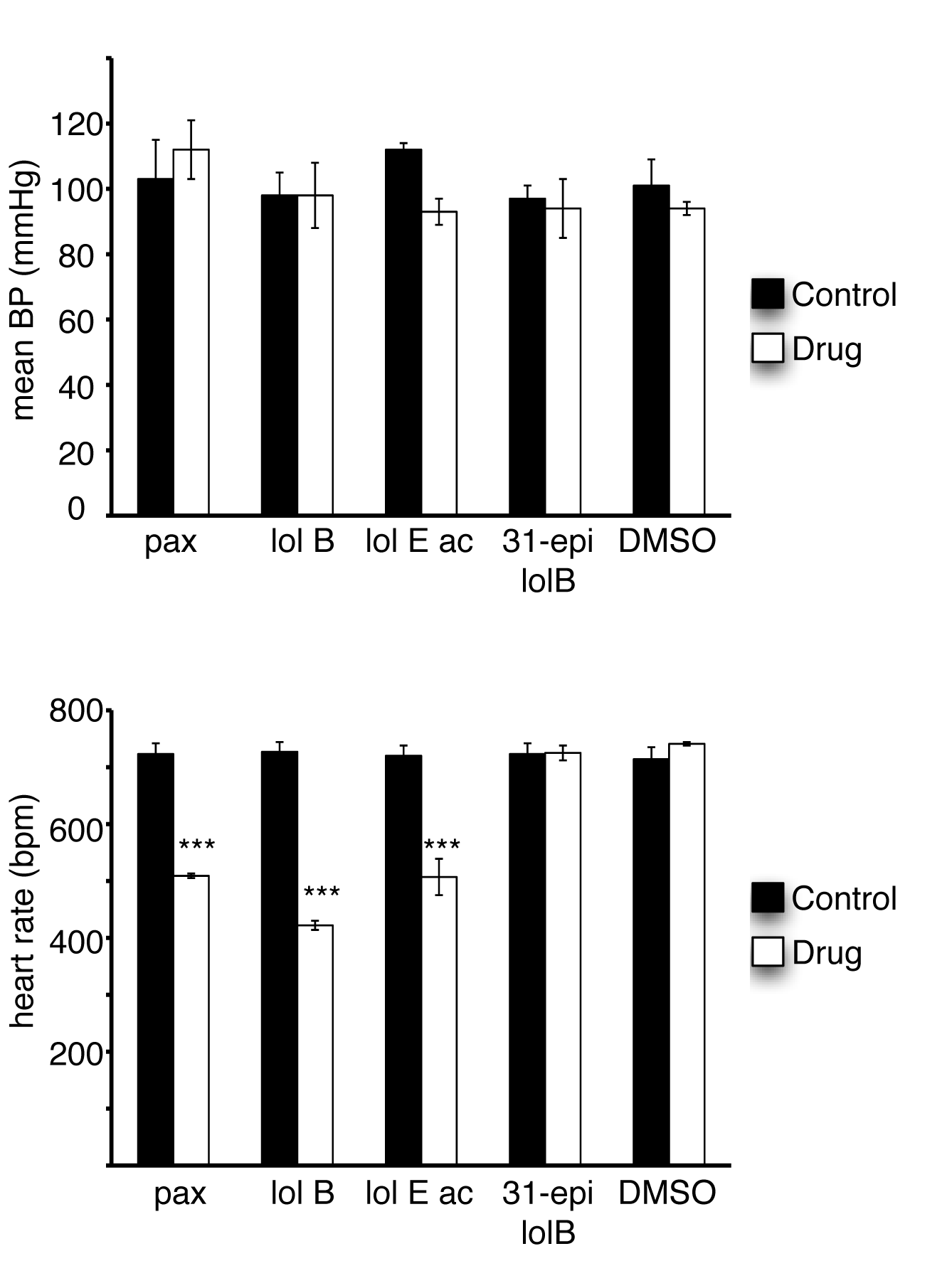

Supplement: Figure S1 — Comparison of peak effect of indole diterpene compounds on heart rate and blood pressure in wild-type mice. The effect of 8 mg/kg paxilline (pax), 4 mg/kg lolitrem B (lol B), 8 mg/kg lolitrem E acetate (lol E ac), 20 mg/kg 31-epilolitrem B (31-epilol B), and DMSO controls, in wild-type mice on: (A) heart rate, and (B) blood pressure. Significance was tested using least significant difference post hoc test after analysis of variance. All data are mean ± S.E.M. *** P<0.001. (4.85 MB TIF) [file pone.0008698.s001.tif]
